# Reframing the Science and Practice of Spinal Cord Injury Rehabilitation: Two Decades of Reflection

**DOI:** 10.46292/1945-5763-29.suppl.iv

**Published:** 2023-11-17

**Authors:** B. Catharine Craven, Milos R. Popovic, Kristin E. Musselman, Curtis Black, Heather Dow

Anniversaries are a time for pause, reflection and celebration. The Canadian Spinal Cord Injury – Rehabilitation Association (CSCI-RA) was born in concept following a discussion among colleagues over lunch in Toronto on a crisp fall afternoon with leaves falling around us in 2002. We animatedly discussed the need for, and value of, interprofessional dialogue among clinicians and scientists with persons with spinal cord injury (SCI) and families to bring about change in the field of SCI rehabilitation. At that time, I had no idea when I committed to host a national SCI rehabilitation meeting that I would be actively engaged in the organization and its mandate two decades later.

**Figure 1.**
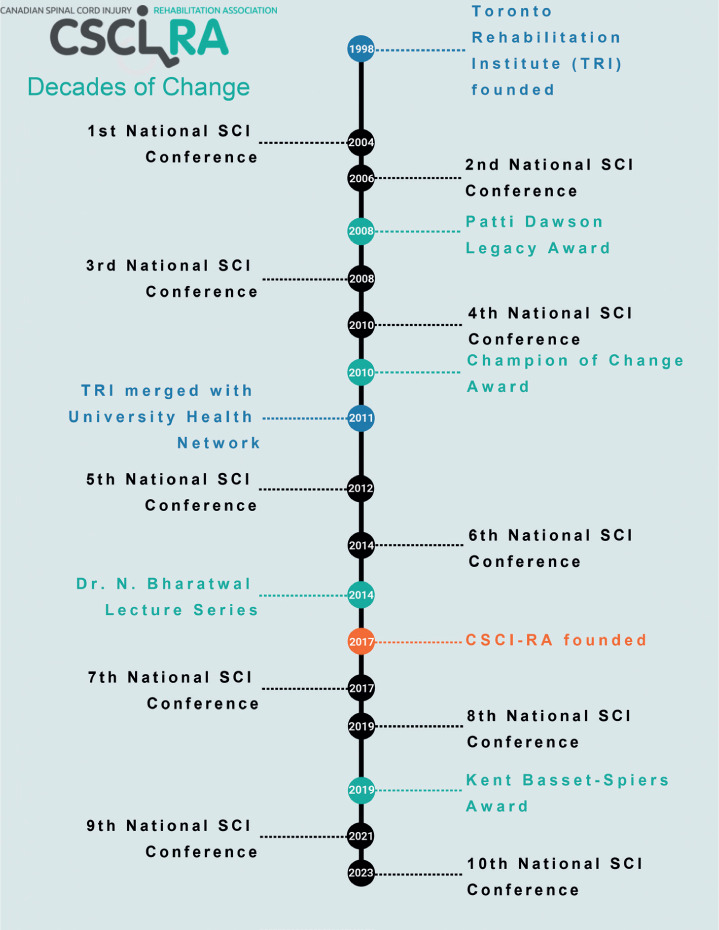
Timeline of the National SCI Conferences and the CSCI-RA.

During the growth and development of our organization (see **Figure 1**), I am proud to say that from the outset we have engaged people living with SCI, family care providers, and stakeholders across the country to foster a culture that promotes comradery and dialogue with staff, scientists, and regulated health care providers. We initiated having 25 to 30 people with lived experience from across the country attend our meetings, long before engagement of partners with lived experience was a concept! We have had many memorable moments and gatherings of staff, students, sponsors, and people with lived experience for photos and wheelchair races in the exhibit halls over the years.

From the outset, we have relied heavily on volunteerism. We are a small organization with a growing membership. We do not have a regular revenue stream or membership reserves to call upon; each conference is its own “special project” from a financial and logistical perspective. We have benefited from group momentum and have leveraged partnerships with many organizations to realize our vision. The initial funds to support the creation of CSCI-RA were provided from Barry Munro and the Canadian Spinal Research Organization, with many matching investments thereafter. Partner organizations have included KITE Research Institute, University Health Network Foundation, Spinal Cord Injury Ontario, Ontario Neurotrauma Foundation, Praxis Spinal Cord Institute (former Rick Hansen Institute), the Canadian Association of Physical Medicine and Rehabilitation, Wings for Life, Craig H Nielsen Foundation, March of Dimes, *Journal of Spinal Cord Medicine*, The International Spinal Cord Society, The Academy of Spinal Cord Injury Professionals, Inc. (ASCIP), and the American Spinal Injury Association (ASIA). These organizations have helped to lift us up and buoyed our efforts at many critical time points in our history. SCI Ontario has been a stalwart partner in enabling people with lived experience in Ontario and across the country to participate through supporting attendant care needs and the orientation and debriefing of people with lived experience before and after meetings. We owe a debt of gratitude to sustaining corporate sponsors who have supported us from concept to reality and who have helped to maintain a learning culture by supporting educational offerings for members between the biannual conferences and annual general meetings.

Reflecting over the years, I am proud of our commitment to address and talk about issues that make us all uncomfortable—everything from the wonders of neuroanatomy to inequity in health services for women and veterans through a reconception of how we evaluate our success. We have frankly discussed gaps in knowledge, care, research, and resources from a variety of perspectives.

Spinal cord injury remains a complex and relatively rare disease among Canadians.^1^ Although, with the rise in incidence of nontraumatic SCI, the proportion of 40 million Canadians requiring SCI rehabilitation services is likely to continue to expand, such that it will not be who can but rather who is most likely to benefit from rehabilitation services. With the recent advent of guidelines,^2^,^3^ best practices, indicators of quality care,^4^-^15^ collaborative networks and implementation science,^16^ and a registry,^1^ one can envision a future where precision rehabilitation^17^ is a viable future entity.

Canada is a large country spanning 9.98 million km^2^; 5514 km from east to west and 4634 km from north to south.^18^ We continue to be challenged by geography as we try to provide tertiary, and sometimes quaternary, SCI rehabilitation services to people with SCI living in the community, many who live more than 150 km from their regional or provincial rehabilitation centre. Despite the geographic and physical barriers to care, we continue to support a vision for interprofessional delivery of in-person SCI rehabilitation with remote monitoring and transitions to local community services where appropriate.^19^,^20^ The pandemic provided an opportunity to accelerate our virtual educational and social support offerings to members throughout the country and to conduct our annual general meetings. Despite these advances in care delivery, we continue to value the opportunity to meet in person to exchange perspectives, ideas, and values on a biannual basis.

Throughout the history of CSCI-RA events (see **Figure 1**), we have openly discussed our failings as a community, included the voice of people with lived experience, featured the work of junior and evolving mid-career scientists (see **Table 1**), discussed hot topics in the form of “Top 6 Articles You Should Have Read,” and celebrated the growth and development of the field with the creation of new awards and expansion of abstract award categories over time. We have embedded student social and speed-mentoring sessions into our biannual meetings to support the growth and development of the field. Many former student abstract winners are now leaders in the field of SCI rehabilitation. These efforts have helped to foster a sense of community, which is palpable among conference attendees; we have invited others to comment and provide feedback through special issue publications associated with our meetings.

**Table 1.** National SCI conferences, themes, and keynotes

**Conference Date**	**Theme**	**Keynotes**
**1st conference 2004 Sep 17-18**	The Evolving Architectureof Patient Care, Education, and Research	Jeff Blackmer – Living Wills Jane Hsieh – Fampridine: A Canadian Story Mark A. Ware – Effects of Cannabis on Neuropathic Pain
**2nd conference 2006 Oct 26-28**	The Evolving Architectureof Patient Care, Education, and Research	R. Lee Kirby – Optimizing Wheelchair Safety and Performance Through Wheelchair Skills Training Armin Curt – Spinal Cord Plasticity: Implications for Spinal Cord Injury Rehabilitation Charles H. Tator – What Regenerative Strategies Exist and Are in Development That Will Be Applicable for Chronic Spinal Cord Injury Patients? Susan Harkema – Locomotion Training for Recovery After Spinal Cord Injury: The Evidence, the Myths, and the Reality Diana D. Cardenas – Clinical Trial Designs for Spinal Cord Injury Research
**3rd conference 2008 Nov 6-8**	The Evolving Architectureof Patient Care, Education, and Research	Brian K. Kwon – Translational Research in Spinal Cord Injury: Clinical Trials and Tribulations John Steeves – Experimental Treatments for Spinal Cord Injury: The Devil Is in the Details or Lack Thereof John Shepherd – The Future of SCI Is Diabetes? Luc Noreau – Quality of Life From a “Fashioned Concept” to a Conceptual and Operational Way for Use as an Outcome Indicator in SCI Research and Service Delivery Serge Rossignol – Approaches to Study and Promoting Recovery of Function after Spinal Cord Injury
**4th conference 2010 Oct 29-30**	Linking Research to Practice	David S. K. Magnuson – Loss and Gain of Function – Activity Dependent Plasticity After SCI Clifford J. Rosen – Muscle – Bone – Fat Interactions Daniel E. Graves – All Limbs Lead to the Trunk John Flannery – Clarity During Challenging Conversations Joanne Smith – Nutrition, The Missing Link
**5th conference 2012 Oct 18-20**	Translating Neural Engineering and Novel Therapies	Jaynie Yang – Retraining Walking After SCI to Induce Neuroplasticity and Useful Walking Mark S. Nash – SCI: Evidence-based and Heuristic Approaches to Customization of Care for Cardiometabolic Syndrome Keith Tansey – Profiling Motor Control in SCI: Moving Towards Individualized Therapy and Evidence-based Care Progression Brian K. Kwon – Biomarkers in Acute SCI – A Translational Imperative Carl Hiebert – Gift of Wings
**6th conference 2014 Oct 3-4**	Bioinformatics Inform SCI Rehab	Amy K. Wagner – Rehabilomics – A Conceptual Framework for Personalized and Translational Rehab Care Douglas J. Weber – Neural Engineering Science and Tech for Restoring Function to Damaged Neural Circuits Nicole Mittmann – How to Win Friends and Influence People: The Role of Health Economics in Decision Making Gale G. Whiteneck – Turning Data into Information: Lessons from SCIRehab Kim Anderson-Erisman – Effectively Utilizing SCI Consumer Input to Guide Research
**7th conference 2017 Nov 9-11**	Military Medicine & SCI Rehabilitation – Novel Intersections	Brian Hodges – Without Compassion There Is no Healthcare Gordon Mitchell – Intermittent Hypoxia and Neurorecovery John F Ditunno, Jr. – Linking SCI Rehabilitation Between Wars: The Deaver-McKenzie Legacy Cindi M. Morshead – Neural Stem Cells: From Basic Biology to Tissue Repair Corporal Michael Clarke – A Life Lived with Spinal Cord Injury Daniel Rogers – Effects of Transcutaneously Modulated Spinal Networks on Sensory-Motor Function Post-paralysis William Geisler – Setting the Stage
**8th conference 2019 Oct 17-19**	Sex, Gender, and the Health of Women	Angela Colantonio – Sex and Gender Considerations in Neurotrauma Research Lisa Boivin – Image-based Storytelling: Painting the Path of Indigenous Resilience Stacy Elliott – Sexual and Fertility Rehabilitation in Women Following SCI Amie B. McLain - #nevertheless she persisted…again. The Struggle for Women with SCI to Obtain Reproductive Health Care
**9th conference 2021 Nov 16-18**	Innovations in Care	Jill Cameron – Family Caregiving Research: Reflecting on the Past to Inform the Future Monica Perez – Strengthening of the Corticospinal Pathway After SCI John Shepard – Identifying Cases of SCI/D in a Database of Primary Care Electronic Medical Records Robert Riener – Robot-aided Therapy of the Upper and Lower Limbs

The special issue of the *Journal of Spinal Cord Medicine* from 2012 to 2021 was integral in demonstrating that Canadians are leaders in SCI rehabilitation health service delivery. Articles associated with these five prior issues^21^-^25^ have been viewed (views and downloads) 94,726 times to date, with each issue averaging approximately 20,000 views as of July 2023.

Over the years, we have had external parties donate funds to create awards to honor patient advocates (Patti Dawson Advocacy Award, see **Table 2**), long-serving clinicians (Dr. Nirmala Dilip Bharatwal Lecture Series), research excellence (Kent Basset Spiers Award, see **Table 3**), and the Champion for Change Award (see **Table 4**). The lists of prior awardees reflect the depth and variety of Canadian SCI rehabilitation expertise among students, staff, advocates with lived experience, and scientists. You will note that many former student awardees are now faculty at leading organizations around the world.

**Table 2.** Patti Dawson Activist of the Year Awardees

**Award Criterion**: Goes to a SCI-O activist who spends the most hours per year in advocacy. It acknowledges individuals who have made a personal commitment to pursuing a career focused on SCI advocacy, either through advocacy research or promoting system change; recognized, assisted, and rewarded for their humane efforts towards the betterment of others living with SCI
**Awardees**
Anita Kaiser
Blair Williams
Nicholas Schoenoff
Maayan ZIv
Wendy Murphy
Chris Stigas
Dave Shannon
John Shepherd
David Onley (Posthumously)

**Table 3.** Kent Basset Spiers awardees

**Award Criterion**: Recognizes an outstanding individual who has demonstrated long-term, influential commitment to people with spinal cord injuries, including research, industry achievement, health care, and/or community service to the spinal cord injury community	
** Year**	**Awardee**
2021	Kent Bassett Spiers
2023	Kristine Cowley

**Table 4.** Champion for Change awardees

**Award Criterion**: Acknowledges an outstanding individual who has changed the fabric of the SCI community by enhancing patient care and augmenting research production and implementation, through sustained and exemplary leadership			
**Year**	**Conference**	**Awardee**	**Institution**
2010	4th	Charles Tator	University Health Network, University of Toronto
2012	5th	John Steeves	International Collaboration on Repair Discoveries(ICORD), University of British Colombia
2014	6th	Serge Rossignol	University of Montreal
2017	7th	Molly Verrier	University Health Network, University of Toronto
2019	8th	Jaynie Yang	University of Alberta
2021	9th	Chester H. Ho	Glenrose, University of Alberta
2023	10th	Colleen O'Connell	Stan Cassidy, New Brunswick

The CSCI-RA is celebrating its 10th National SCI Meeting. A 10th anniversary is typically acknowledged by a gift of aluminum to reflect the strength of our bond and our enduring passion for the field. For those who are able, we ask that you bring any aluminum pop can tabs to the 10th National Meeting for donation to the March of Dimes.

CSCI-RA has been an effective mechanism to raise the profile of SCI rehabilitation in Canada, to help drive academic progress, and to attract students to train and remain in the field throughout their careers. We are grateful to the *Journal of Spinal Cord Medicine* (ASCIP) in the past and *Topics in Spinal Cord Injury Rehabilitation* (ASIA) currently for their support in helping disseminate our annual meeting materials and extend the reach of our organization. Award-winning abstracts and papers from prior special issues have had a high impact, with many views and downloads across North America, Europe, and China. We continue to be inspired by the resilience and accomplishments of people with lived experience and value their vote of confidence and trust when agreeing to evaluate new or novel approaches to care or research. CSCI-RA is our organization (see **Table 5**); we remain open to evolving and are more than willing to collaborate with other like-minded organizations. SCI rehabilitation is an ever-evolving field; we look forward to unleashing the joint efforts of our members, people with lived experience, regulated health professionals, scientists, research staff, collaborative partners, and sustaining sponsors over the next decade. Assist us in making precision rehabilitation a reality!

**Table 5.** CSCI-RA vision, mission, and executive members

**Vision**		
To enhance the recovery, health and wellbeing of all people living with spinal cord injury/disease (“SCI/D”) through research, education and information for patients, families, care providers, regulated healthcare professionals and the scientific community.		
**Mission**		
Foster a community of support and advocacy for comprehensive rehabilitation. Promote and establish standards of excellence for spinal cord injury rehabilitation, Educate members to increase availability of complex services. Foster research in preventing spinal cord injury, improving care, reducing consequences of disability, and finding a cure for both acute and chronic SCI		
**Directors**		
**Role**	**Name**	**City**
CEO	Heather Dow	Kingston, ON
Chair	Dr. B. Catharine Craven	Toronto, ON
Vice-Chair	Dr. Milos Popovic	Toronto, ON
Member at Large	Dr. Kristin Musselman	Toronto, ON
**Officers**		
**Role**	**Name**	**City**
Accreditation	Dr. Colleen O'Connell	St. John, NB
Education	Dr. Hardeep Singh	Toronto, ON
Scientific	Dr. Kristine Cowley	Winnipeg, MB
Student Representative & Mentorship	Dr. Vivian Mushahwar	Edmonton, AB
Abstract Adjudication	Dr. Jose Zariffa	Toronto, ON
Resource Development	Dr. Sukhvinder Kalsi-Ryan	Toronto, ON
Awards	Dr. Cesar Marquez-Chin	Toronto, ON
Journal Liaison	Dr. Julio Furlan	Toronto, ON
